# Additive Manufacturing of Conductive Pathways for Drone Electrical Equipment

**DOI:** 10.3390/polym17111452

**Published:** 2025-05-23

**Authors:** Gintarė Malikėnaitė, Arvydas Rimkus, Darius Rudinskas, Artūras Jukna, Viktor Gribniak

**Affiliations:** 1 Department of Aeronautical Engineering, Vilnius Gediminas Technical University (VILNIUS TECH), Linkmenų Str. 28–4, 08217 Vilnius, Lithuania; gintare.malikenaite@stud.vilniustech.lt (G.M.); darius.rudinskas@vilniustech.lt (D.R.); 2 Laboratory of Innovative Building Structures, Vilnius Gediminas Technical University (VILNIUS TECH), Saulėtekio Av. 11, 10223 Vilnius, Lithuania; arvydas.rimkus@vilniustech.lt; 3 Photovoltaic Technologies Laboratory, Vilnius Gediminas Technical University (VILNIUS TECH), Saulėtekio Av. 11, 10223 Vilnius, Lithuania; arturas.jukna@vilniustech.lt

**Keywords:** 3D printing, electric conductivity, polylactic acid (PLA), experimental measurements, prototyping

## Abstract

Fused filament fabrication is the most common additive manufacturing technology due to its cost-effectiveness and flexibility in customization compared to alternative production techniques. This technology holds significant promise for revolutionizing the design and fabrication of unmanned aerial vehicles (UAVs), also known as drones. The present study continues a research program dedicated to additive drone manufacturing using a simple desktop printer and polymeric materials. The previous investigation in the series determined polylactic acid (PLA) as a potential material for drone fabrication. This research continuation takes a step forward in automating the manufacture of drones, extending the 3D printing concept to producing conductive pathways suitable for electric equipment. The automation reduces the need for manual wire installation in electric equipment. The drone prototype developed in this study demonstrates the feasibility of such automated manufacturing. The analysis of conductive polymeric materials available on the market defines the electrical resistance parameters of additively manufactured UAV components that limit the efficient electric current. The conductivity analysis of the 3D-printed components also determines the materials’ characteristics required to achieve the automated manufacturing goals of the electrically conductive pathways.

## 1. Introduction

Fused filament fabrication (FFF), also known as fused deposition modeling and 3D printing, is the most widely used additive manufacturing (AM) technology, recognized for its cost efficiency and adaptability compared to other production methods [1,2]. The FFF technology has the potential to fundamentally transform the design and production of unmanned aerial vehicles (UAVs), also commonly referred to as drones. The current study advances the research initiative focused on additive manufacturing for drones, utilizing a basic desktop printer and commercially available polymer materials. The first phase of this research identified polylactic acid (PLA) as a viable material for drone construction [3]. This continuation manuscript presents the second phase of the investigation, which advances the automation of drone manufacturing by broadening the 3D printing concept to include the creation of conductive pathways suitable for electronic devices. This fabrication enhancement reduces the need for manual assembly and minimizes errors associated with wiring electronic components.

Conducting polymers (CPs) have garnered significant research attention due to their economic importance, exceptional environmental stability, high electrical conductivity, notable mechanical, optical, and electronic properties, and eco-friendly nature [4]. These characteristics make them applicable in various fields, including energy storage, sensors, electromagnetic interference shielding, photovoltaic devices, and polymeric light-emitting diodes [5]. However, CPs face challenges in AM due to their limited processability, which complicates the control over the electrical conductivity and structural order of pure CPs [6]. Therefore, CPs must be combined with other polymers and additives to achieve suitable conductivity for AM.

Due to their excellent conductivity, metal nanoparticles are the most straightforward choice for ensuring electrical conductivity for polymers, although they are more expensive than carbon-based materials [7]. CP nanocomposites incorporate nanostructured fillers to impart electrical conductivity to otherwise insulating polymer matrices. Huang et al. [8] compared the electrical conductivity of metal and carbon fillers for CPs, finding that the electrical characteristics of carbon nanotubes (CNTs) outperform those of nickel and aluminum but do not reach the properties of copper and silver. Among the available fillers, carbon-based nanomaterials, such as CNTs, carbon black (CB), and graphene, are predominant due to their cost-effectiveness, lightweight nature, and tunable conductivity [6]. These nanofillers establish conductive networks within the polymer through percolation, where electrical pathways form once the filler concentration exceeds a critical threshold [9,10,11]. In particular, CNTs offer exceptional conductivity at low currents due to their high aspect ratio [12]. CNTs have been utilized in advanced AM to enable anisotropic conductivity, enhanced mechanical properties, and sensor functionality [13,14]. However, their high cost, tendency to agglomerate, and processing challenges limit their widespread use, especially in large-scale or cost-sensitive applications.

Alongside advanced carbon-based nanomaterials, cost-effective CB materials have been rediscovered over the past two decades [15,16,17,18]. By comparing the performance of screen-printed electrodes produced using CB, CNTs, and graphene, Cinti et al. [17] emphasized the exceptional electron transfer heterogeneity of CB-based samples compared to the alternatives considered. Estimated at EUR one per kg in 2020 [18], CB’s excellent electrical conductivity [19] makes it a promising option for developing low-cost polymeric structures. Kwok et al. [20] created a conductive polymeric filament by blending CB (15.5–32.3 wt%) with polypropylene. The resulting 3D-printed composite achieved an electrical resistivity of 5 mΩ·m with a percolation threshold of approximately 11.3 wt%, facilitating the production of conductive circuits and sensors. Espera et al. [21] identified percolation thresholds between 1.5 wt% and 3 wt% of CB in selective laser sintering (SLS) polyamide-12 composites, achieving an electrical conductivity of 1.12 mS/m at a 10 wt% CB content.

While carbon-based nanofillers such as CNTs and graphene possess high intrinsic electrical conductivity, their effectiveness in enhancing the electrical conductivity of polymer composites is often limited in practice [22]. This limitation arises due to challenges in achieving uniform dispersion, strong interfacial bonding, and the formation of continuous conductive networks within the polymer matrix [23]. In contrast, due to their superior intrinsic conductivity and better interparticle connectivity, metal fillers typically impart higher electrical conductivity to polymer composites under similar conditions [24,25].

Table 1 summarizes relevant examples from the literature, focusing on the FFF of conductive pathways. The results presented in this table, organized by ascending conductivity, align with the findings of Huang et al. [8], except for the relative inefficiency of multi-walled CNTs. This decrease in CNT efficiency may be attributed to the agglomeration issue of carbon nanoparticles, as noted by Cinti et al. [17]. Furthermore, Table 1 indicates that the conductivity of CB-based 3D-printed pathways is extremely low, which serves as the focus of this study [20,26,27,28,29,30].

This experimental research hypothesizes that FFF technology has the potential to create conductive pathways suitable for drone electrical equipment using commercially available conductive polymers. This fabrication enhancement reduces the need for manual wire installation for electrical equipment. The drone prototype developed in this study demonstrates the feasibility of such automated manufacturing.

## 2. Materials and Research Methodology

This experimental study builds on the research program [3] focused on additive drone manufacturing, utilizing a simple desktop printer and commercially available polymeric materials. The study [3], representing the initial stage of this research program, identified PLA as a viable material for drone construction due to its relatively high mechanical resistance, ease of fabrication, and non-toxicity. In particular, the mechanical PLA strength was substantially (by nearly 30 times) greater than the design stress values. The deformability of FFF plastics, including creep and temperature-induced deformation, may lead to structural issues that warrant further investigation beyond the scope of this study.

This continuation of the research [3] advances the automation of drone manufacturing by expanding the 3D printing concept to create conductive pathways suitable for electronic devices, utilizing PLA materials as previously specified. All specimens were fabricated using a P1S printer (Bambu Lab, Shenzhen, China), equipped with an automatic material system (AMS) for multi-material fabrication and an enclosed build chamber to maintain consistent thermal conditions during the printing process. The AMS unit combined conductive and non-conductive filaments in a single print, enabling automated material switching during fabrication.

The functionality of a drone’s conductive system determines the efficiency criteria of CP. The drone geometry determines the maximum cross-sectional area in this study. Thus, CP conductivity becomes the focus of this investigation, and theoretical calculations are employed to estimate the minimum conductivity required for the AM drone with an integrated conductive system to be functional.

### 2.1. Overview of Commercially Available CP

Several commercially available CP filaments are suitable for 3D printing. Table 2 compares their characteristics, highlighting properties such as material composition, conductivity, 3D printing method, and application [31,32,33,34,35,36]. Like Table 1, this table also organizes filaments by the conductivity specified by the manufacturer. This arrangement visualizes the impact of the filler on both conductivity and the cost of raw materials. In particular, the price of nearly 100 EUR/kg for the CB-based filaments [31,32,33] makes them a promising alternative to highly conductive yet expensive copper particle-based polymers [36] for this prototyping study. Graphene-based materials [34,35] are also costly but do not offer a significant increase in conductivity to justify the financial expense compared to CB-based alternatives.

This study employed a conductive PLA filament [31] containing CB filler. The CP matrix material is the same as that selected for FFF of the drone frame in this study, which mitigates the polymers’ compatibility issues typical of multi-material polymeric composites [37]. The CB additive consists of quasi-spherical carbon particles with a high surface area, enabling efficient electron tunneling and charge transport when uniformly dispersed throughout the polymeric matrix [20]. It can achieve percolation at concentrations as low as 1.5–3 wt% [21]. These CPs achieved bulk resistivities of less than 0.01 Ω·m and are compatible with thermoplastics and FFF technology, making them suitable for printed conductive circuits and low-power electronic elements [19]. The material choice was based on a systematic review of commercially available conductive polymer filaments, ensuring the achievable conductivity within the practical constraints of procurement and regional availability. In particular, a more conductive alternative [33] in Table 2 was not available to purchase during the test preparation. Still, the choice of CP does not affect the research steps and methodology in this investigation, and the conclusions can be extended to apply to the alternative CPs listed in Table 1 and Table 2.

### 2.2. Conductive Specimens

The test results [20,38,39] are analyzed to determine the design of the CP circuit samples. Kwok et al. [20] used both MakerBot Dual (MakerBot, New York, NY, USA) and FlashForge Creator (Flashforge, Zhejiang, China), and Zhang et al. [38] employed the MakerBot Replicator 2 printer (MakerBot, New York, NY, USA). On the contrary, Flowers et al. [39] opted to build a custom 3D printer based on the design of the open-source D-Bot printer [40] due to the limitations of suitable commercially available apparatuses for dual-material printing. Table 3 describes the essential settings of these studies.

**Figure 1:**
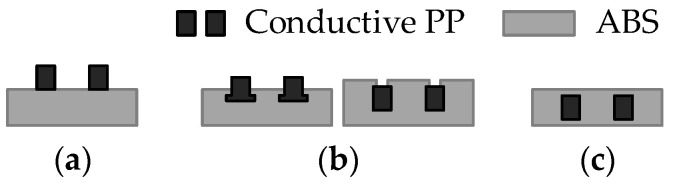
Schematics of PP circuits in ABS substrate [20]: (**a**) non-encapsulated; (**b**) partially encapsulated; (**c**) entirely encapsulated.

Kwok et al. [20] explored the conductive pathway delamination problem, considering the sample geometries shown in Figure 1. The analysis revealed that without encapsulation (Figure 1a), delamination occurs readily through peeling, while both partial encapsulation (Figure 1b) and complete encapsulation (Figure 1c) prevent delamination. In the present study, the adhesion between ordinary and conductive polymers is ensured due to the compatibility of the matrix materials (Section 2.1). Therefore, the “open” configuration (Figure 1a) is used for the conductivity measurements to monitor electrical characteristics throughout the conductor’s depth. The drone prototypes utilize both non-encapsulated and partially encapsulated conductive pathways (Figure 1a,b) to facilitate visual inspection of the fabrication quality. The entirely encapsulated pathways (Figure 1c) are anticipated for the functional FFF drones.

**Figure 2:**
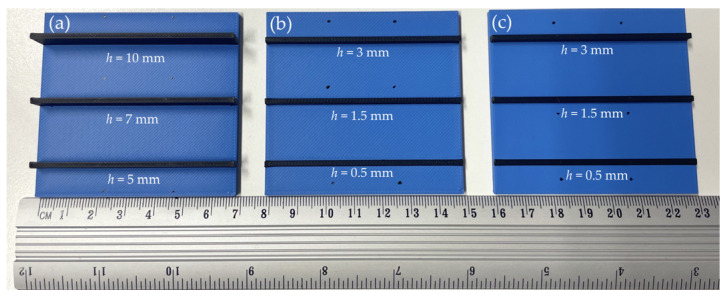
Conductivity measurement specimens: (**a**,**b**) printed using 0.20 mm layer thickness and (**c**) using 0.08 mm layer thickness.

Figure 2 shows the specimens used for the conductivity measurements in this study. The conductive samples have a fixed length of 70 mm and a width of 2 mm, while the cross-section height was systematically increased to evaluate its impact on electrical conductivity. The pathways have the following heights: 0.5 mm, 1.5 mm, 3 mm, 5 mm, 7 mm, and 10 mm. The models were processed for printing using the Bambu Studio software (Bambu Lab, Version 1.10.1.50, Shenzhen, China). Each model was configured for dual-material fabrication, employing a 1.75 mm non-conductive PLA filament (blue) as the structural base and ProtoPasta Electrically Conductive Composite PLA [31] (black) for the conductive tracks. To ensure structural integrity and reliable electrical performance, the slicing parameters included 100% infill density and two outer wall loops, providing smooth surface finishes and precise dimensional accuracy. Print speeds were set to 200 mm/s for outer walls and 230 mm/s for infill, balancing print quality and fabrication efficiency. All specimens were printed using the P1S printer (Bambu Lab, Shenzhen, China), with a 0.4 mm nozzle and a textured build plate. The printer’s AMS facilitated seamless, automated transitions between structural and conductive materials, eliminating the need for manual intervention during fabrication. The extrusion temperature was consistently maintained at 220 °C, while the print bed was heated to 55 °C to minimize warping and ensure optimal adhesion.

The conductive specimens were fabricated with two distinct layer thicknesses—0.20 mm (Figure 2a,b) and 0.08 mm (Figure 2c)—to evaluate the influence of print resolution on the overall quality and functionality of the specimens. It is assumed that both geometrical parameters remain uniform throughout the length of the sample. For a sample width *b* = 2.0 mm and a height *h* varying between 0.5 mm and 10 mm, the resulting cross-sectional area yields a current density of approximately 10 A/m^2^ to 40 A/m^2^ when a direct current is applied over the variable sample length in this study. Under these conditions, the associated electric power dissipation in the 70 mm long samples remains below 100 µW, implying a negligible self-heating effect and, consequently, an insignificant variation in the material’s resistivity due to the applied bias current.

**Figure 3:**
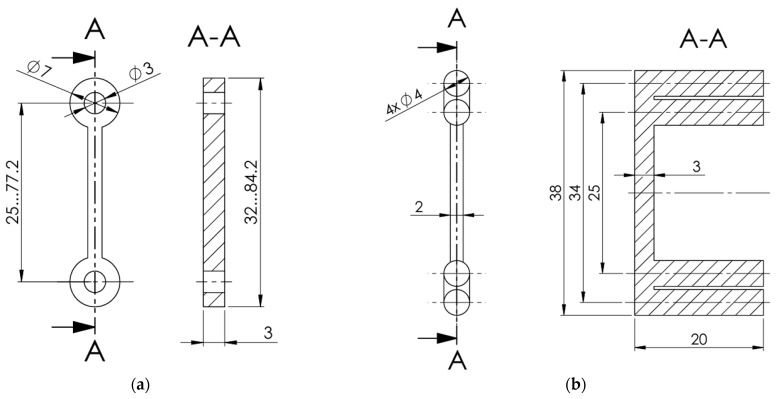
Schematic drawing of the 3D-printed conductive track design with (**a**) bolted and (**b**) wrapped wire connections. Note: dimensions are presented in millimeters.

This study also examines several designs of the contacts between the prototype specimens and the power supply and receiver: mechanical (bolted), wrapped, and direct contact without additional wires. The first two contacts were used for the conductive prototypes (Figure 3), whereas the last one was applied in the drone prototype.

The bolted contact provides a straightforward reference, while the wrapped alternative reflects the fabrication method of mechanically and electrically stable connections outlined in the MIL-STD-1130C standard [41], which specifies requirements for solderless wrapped electrical connections. The National Aeronautics and Space Administration (NASA) also endorses wire-wrapping for specific applications [42]. According to NASA standards, this technique ensures a gas-tight joint, as each 90° turn around the square post fractures the wire’s oxide layer, promoting a stable connection (Figure 3b). This method provides a stable, electrically efficient connection that resists vibration and mechanical stress, minimizing resistance fluctuations and ensuring long-term durability. Using wrapping techniques around posts helps improve contact reliability, essential for maintaining stable electrical performance in drone applications.

**Figure 4:**
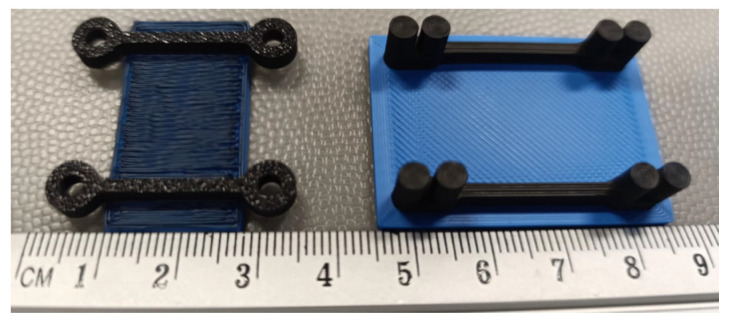
3D-printed conductive pathways with bolted (**left**) and wrapped (**right**) wire connections.

Figure 4 shows the 25 mm long conductive pathways fabricated with bolted and wrapped wire connections (without wires). All these specimens were modeled and manufactured using the Bambu Studio slicing software and a P1S printer, with the same materials and printing settings as those used for the specimens shown in Figure 2. As in the previous specimens (Figure 2), the elements, shown in Figure 4, utilize the PLA (blue) plate as a fixing base to ensure the stability of geometry and measurement. To ensure fabrication quality, the specimens with bolted connections were produced in a bottom-up position, resulting in the base plate’s darkest color, as shown in Figure 4.

### 2.3. Conductivity Assessment Methods

Electrical conductivity determines the efficiency criteria of CP in this study. Its measurement methods depend on the geometry of the 3D-printed structure and the necessary precision. Blachowicz et al. [43] classified the following techniques as the most suitable for the FFF printed pathways:

- The two-point probe method is extensively employed for determining electrical resistance due to its procedural simplicity and minimal instrumentation requirements. This technique introduces an electric current through two probe-specimen interfaces and conducts through the test material. According to Ohm’s law, the voltage drop measured across the probes comes from the combined effect of the material’s intrinsic (bulk) resistance and the interfacial contact resistances at the probe–material junctions. This method yields reliable and accurate resistance measurements only under specific conditions: (a) when the probe material contact regions are sufficiently confined, ideally approximating point contacts, thus promoting a linear and well-defined current–voltage (*I*–*V*) characteristic; or (b) when the contact resistances are negligibly small relative to the bulk resistance of the material, typically by several orders of magnitude, such that their influence on the total measured resistance can be considered insignificant.
- The four-point probe method effectively mitigates the limitations inherent to the two-probe method, particularly those stemming from contact resistance at the probe–material interfaces. In this measurement configuration, a controlled current is introduced through the two outer electrodes, while the resulting voltage drop is sensed independently by the two inner electrodes. This spatial decoupling of current injection and voltage measurement eliminates the influence of contact resistance at the voltage-sensing electrodes, as these probes conduct an insignificantly small current. Consequently, the measured voltage only reflects the intrinsic electric potential of the test sample, allowing higher precision in determining the electrical resistance.

Kwok et al. [20] tested both the two-point and four-point measurement methods, finding that the contact resistance was minimal (±2%) compared to the total resistance. Consequently, the two-point method was adopted for all subsequent electrical measurements. Zhang et al. [38] and Flowers et al. [39] utilized the two-point method exclusively to measure conductivity. Kwok et al. [20] and Flowers et al. [39] applied silver ink (Electron Microscopy Sciences, catalog number: 12630) to small areas (width: 1–2 mm; length: 3–4 mm) at the ends of the samples to diminish the contact resistance between the measuring probes and the conductive thermoplastic composite. This study employs the four-probe technique to evaluate the electrical resistance of the samples (Figure 1a).

**Figure 5:**
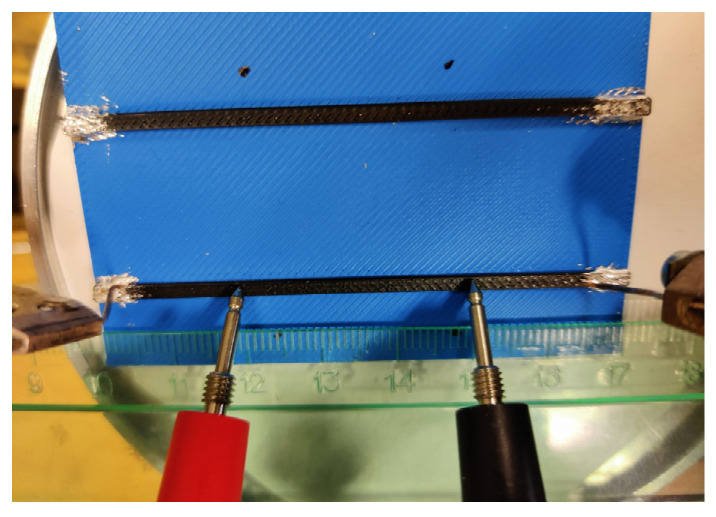
The four-point probe setup for 3D-printed conductive tracks.

Surface roughness should also be considered when measuring the resistance of conductive polymers. Commercial electrodes are not designed for uneven or soft surfaces and may struggle to establish reliable contact with 3D-printed pathways [44]. A typical solution to this issue involves applying a layer of silver paint to the surface, creating a more dependable contact point and ensuring proper electrical connectivity [45]. This approach mitigates the impact of surface irregularities and enhances the accuracy of the testing results. Therefore, this study utilizes an eutectic indium–gallium (In-Ga) alloy paste to ensure a uniform current distribution across the entire cross-sectional area of each stripe-like sample. Due to its eutectic composition, the In-Ga paste remains liquid under ambient conditions, allowing it to closely conform to the surface morphology of the fabricated samples. Both terminal faces of each sample were coated with the In-Ga paste, ensuring complete coverage of the cross-section, as shown in Figure 5. This configuration enables an almost homogeneous current density throughout the sample and establishes a stable, low contact resistance between the probe and the test material.

The voltage measured between the inner probes is then given as

(1) $$V=I\cdot R=I\cdot \rho \cdot \frac{L}{A}=I\cdot \rho \cdot \frac{L}{b\cdot \;h}\;$$

where *ρ* and *R* denote the electric resistivity and resistance of the sample; *L* is the distance between the inner voltage-sensing probes; *A*(=*b*·*h*) represents the cross-section area of the conductive pathway; and *b* and *h* are the sample width and height. The resistivity of the conductive tracks was calculated using the following formula

(2) $$\rho =\frac{1}{\sigma }=\frac{R\cdot A}{l}$$

where *σ* and *l* are the conductivity and length of the conductive track.

The electrical resistivity of the printed conductive pathways (Figure 2) is measured using the two-point probe and four-point probe methods. The first method utilizes a universal VC505 multimeter (Orangjo, Shenzhen, China). The four-point probe measurement setup applies a 100 µA current at two outer contact points; the inner probes monitor the resulting voltage drop. A Keithley 2400 SourceMeter (Keithley Instruments, Solon, OH, USA) supplies the current, and voltage measurements are conducted using a Keithley 2002 multimeter (Keithley Instruments, Solon, OH, USA). To improve electrical connectivity and mitigate the uncertain effects of surface roughness in the printed samples, the electrode contact points were coated with an In-Ga paste, as shown in Figure 5.

At the first measurement stage, the four-point probe measurements in Figure 5 were conducted repeatedly, using distances of 10 mm, 15 mm, 20 mm, 25 mm, 30 mm, and 35 mm between the internal electrodes, with the electrodes placed at the top surface and side surface near the bottom contact with the base (blue) PLA. Thus, 36 measurements were achieved per conductive pathway. Hence, two groups of 36 measurements were created for the test samples shown in Figure 2a,b. The first group compares the average resistivity obtained for different electrode distances (Figure 5). The second group includes resistivity measurements of the top and bottom layers, specifically regarding the direction of the conductive pathway. Additionally, the third group consists of 18 measurements of the resistivity of the specimens fabricated with a 0.08 mm layer thickness (Figure 2c), varying the distances between the electrodes. This group is an alternative to the first group, estimating the effect of building layer thickness on the pathway’s resistivity.

**Figure 6:**
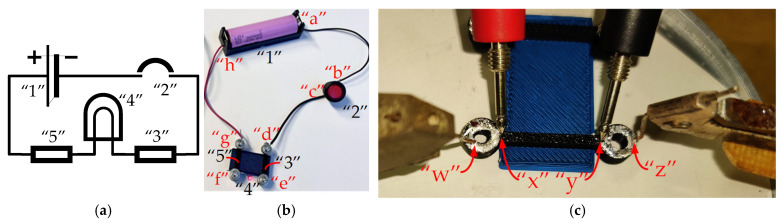
The resistance measurement example employing the 25 mm long conductive pathway with bolted wire connections: (**a**) the principal electric scheme; (**b**) active circuit; (**c**) the four-probe measurement setup of the conductive pathway.

The second measurement stage employs the active circuit shown in Figure 6 to evaluate the electrical performance of the printed conductive tracks. The circuit consists of a power source (Li-ion battery type 18650) “1”, a 3 mm red LED “4” with an operating current of 2 mA, and two conductive tracks (“3” and “5”) with bolted wire connections. These tests employ the four-probe technique, where a Keithley 2002 multimeter (Keithley Instruments, Solon, OH, USA) was used to measure voltage drops across various segments of the active circuit. Two different conductive lengths, i.e., 25 mm and 77.2 mm, were considered, and Figure 6 shows the 25 mm long sample as an example. Figure 6a shows the principal electrical scheme of the circuit. The battery voltage was measured between contact points “a” and “h” indicated in Figure 6b; the voltage across each conductive pathway was recorded between points “d” and “e” (track “3”) and “f” and “g” (track “5”); the LED voltage drop was determined between points “e” and “f.” Following these measurements, the circuit was deactivated using the switch button “2”, and the Keithley 2002 multimeter was connected at points “b” and “c” to measure the total current of the circuit. The resistance values were calculated based on Ohm’s law. These values are presented in the “Total” column of Table 4.

The circuit was then disassembled by removing the bolts, and each conductive track was evaluated independently using the four-probe method, as shown in Figure 6c. The In-Ga paste was applied to the track ends to reduce interfacial resistance. A direct current (DC) of 100 µA was supplied using a Keithley 2400 SourceMeter through points “w” and “z” (Figure 6c), and the DC voltage drops were measured between points “x” and “y” by the Keithley 2002 multimeter. These measurements were repeated three times per track to ensure the consistency of the results. The resulting voltage and current data were used to calculate the resistance of each conductive pathway. These values are presented in the “Track” column of Table 4. Finally, the contact resistance at each bolted interface (points “d”, “e”, “f”, and “g” in Figure 6b) was determined by subtracting the intrinsic track resistance (the “Track” column of Table 4) from the “Total” resistance. Table 4 summarizes the resultant values in the “Contact” column. Table 4 also describes the resistivity of the printed pathway estimated by Equation (2) in the last column.

### 2.4. Heating Tests

Kwok et al. [20] also reported the effect of temperature on the resistance of the conductive pathways. Thus, this study also investigates the residual conductivity of the 3D-printed polymer after it has been heated. Hence, the 25 mm long specimens were exposed to 60 °C for 1 h in a heating chamber (Zyle ZY100FD, Krinona Ltd., Kaunas, Lithuania) to assess the thermal stability and residual conductivity of the 3D-printed tracks. Three specimens of both connection types (i.e., Figure 4) were tested. Before heating, the initial resistance of each specimen was determined utilizing the Orangjo VC505 multimeter. The measurements were repeated twice: (1) immediately after the heating, and (2) after cooling to 20 °C for an hour.

Guadagno et al. [46] found that electrical anisotropy leads to current-induced heating in 3D-printed pathways. Therefore, this test program also measured the electricity-induced heating from the electrical induction through the printed structure. Two circuits utilizing the bolted wire connection with 25 mm and 77.2 mm conductive tracks were evaluated under controlled thermal conditions in a custom-built chamber (Daikra, Klaipėda, Lithuania). Before the tests, the deactivated specimens were stored in a thermal chamber at 15.0 °C and 58% relative humidity for 24 h to stabilize the temperature distribution within the specimens. After that, the circuits were switched on and continuously operated under the same environmental conditions. Thermal images were captured at 10-min intervals over a 70-min period using a FLIR E60BX thermal imaging camera (Teledyne FLIR, Wilsonville, OR, USA). This camera features an uncooled microbolometer focal plane array with an infrared resolution of 320 × 240 pixels and a thermal sensitivity (NETD) of less than 0.045 °C at 30 °C, enabling the precise detection of subtle temperature differences. The device operates within a temperature measurement range of −20 °C to +120 °C, with an accuracy of ±2% of the reading (for ambient temperatures between 10 °C and 35 °C). The camera was mounted on a fixed tripod approximately 0.5 m from the specimens to ensure consistent imaging geometry throughout the test.

### 2.5. The Illustrative Prototypes

The Nano Long Range drone [47] was selected for its compact size, lightweight design, and ease of use. It is a compact quadcopter designed for a 1–2 km flight range. The drone weighs approximately 95–97 g and features a 135 × 135 × 22 mm^3^ frame. Powered by a single 18650 Li-ion battery type, rated at 3.6 V nominal voltage, it benefits from the high energy density and low weight typical of this cell type. The battery supports flight durations of 10–15 min, depending on throttle use and conditions. The drone utilizes four Flywoo 1202.5 11,500 kV brushless motors (Flywoo, Shenzhen, China), operating at 3.7 V with a current draw of approximately 1.15 A at 50% throttle and 4.9 A at 100% throttle. These motors are paired with Gemfan 3018 two-blade propellers (Gemfan, Ningbo, China), optimized for efficient thrust in lightweight builds. The reference [47] also includes an STL file with the precise drone geometry, which helps avoid modeling errors.

**Figure 7:**
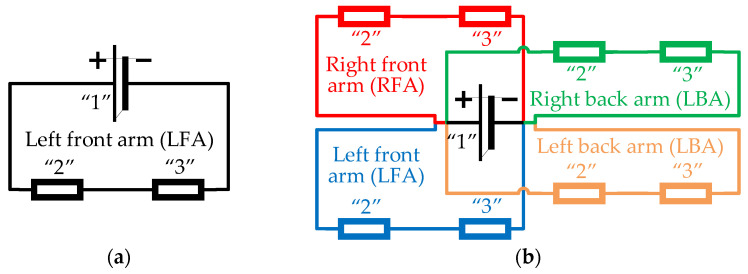
The principal electric schemes of the drone prototypes: (**a**,**b**) Prototypes 1 and 2. Note: “1” = battery; “2” = 3D-printed conductive track; “3” = active circuit component (e.g., LED, motor).

**Figure 8:**
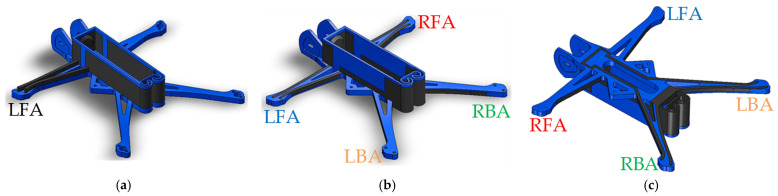
SolidWorks 3D models of the Nano Long Range drone prototypes incorporating integrated conductive pathways: (**a**) Prototype 1; (**b**,**c**) top and bottom views of Prototype 2. Note: Conductive elements (black) and structural components (blue); the arm notation corresponds to Figure 7.

This study develops two simplified drone prototypes utilizing a Li-ion battery type 18650. The geometry and dimensions of the drone’s frame remain consistent with the Nano Long Range drone [47] for both models. Prototype 1 represents the drone model with a single active circuit, serving as the basis for the theoretical analysis of circuit capacity. Prototype 2 features a more practical arrangement of the conductive pathways, ensuring the functionality of four active components. Due to the limitations of the conductive polymer, the drone’s motors were replaced with LEDs to lower power demands. Figure 7 shows the principal electric schemes of these drone prototypes. Figure 8 shows the 3D models of both drone prototypes created using SolidWorks software (Educational Edition 2023 SP2.1, Dassault Systèmes, Vélizy-Villacoublay, France).

**Figure 9:**
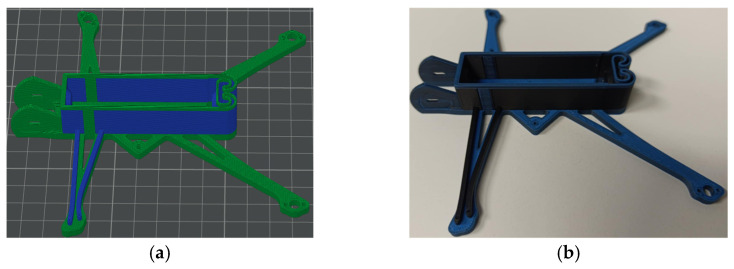
Manufacturing of drone prototype: (**a**) sliced model (the blue color shows the conductive polymer); (**b**) fabricated prototype (the conductive polymer is black).

**Figure 10:**
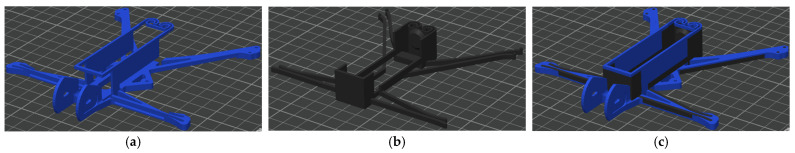
Sliced model of the drone Prototype 2: (**a**) non-conductive PLA frame; (**b**) conductive pathways’ 3D network; (**c**) assembled model prepared for fabrication.

Figure 9 and Figure 10 present the fabricated prototypes and the models prepared with the Bambu Studio software for 3D printing. Both drone prototypes incorporate a 2 × 3 mm^2^ cross-section of the conductive pathways. In Prototype 1 (Figure 9), the conductive paths were formed on the top of the front arm without compromising its structural integrity. On the contrary, Prototype 2 (Figure 10) has conductive pathways integrated into the frame structure, preserving the drone’s external shape and accommodating the dimensional constraints of its arms, which are 4 mm in height and approximately 5 mm wide at their narrowest sections. This case exemplifies the additive manufacturing possibilities for forming integrated, conductive pathways that can entirely replace metal wires and contacts. In the latter model, the composite frame’s mechanical resistance has been outside the scope of this study, forming a topic for further research. A custom-designed battery holder was fabricated entirely from the conductive polymer, eliminating all metal components from the drone’s electrical system and demonstrating the feasibility of fully 3D-printed circuitry.

The drone prototypes were fabricated using the same conductive and non-conductive materials, slicing parameters, and dual-material printing methodology detailed in Section 2.2, ensuring consistency in manufacturing conditions and conductivity performance. Thus, drone Prototype 1 (Figure 8a) utilized 17.61 g of basic PLA (blue) and 7.28 g of conductive plastic (black), for a total weight of 24.89 g. Prototype 2 (Figure 8b,c) has a total weight of 24.59 g, comprising 14.43 g of basic PLA and 10.16 g of conductive PLA materials. Dual-material printing involves additional filament consumption for purging during material changes to prevent cross-contamination between polymers. This extra material consumption, similar for both prototypes, is not reflected in the reported weights.

## 3. Results and Discussion

### 3.1. The Manufacturing Settings Effect

The 70 mm long specimens (Figure 2b,c) illustrate the influence of building layer thickness on the resistivity of the conductive pathways. At a layer thickness of 0.20 mm, the resistivity remains relatively consistent across all samples, independent of the height of the conductive sample, with a standard deviation of less than 2.6%, as determined from 36 measurements that varied the distances between the electrodes (Figure 5). In contrast, samples printed with a layer thickness of 0.08 mm exhibit higher scatter, with resistivity deviations reaching up to 6.4%. Moreover, these samples show a slightly higher average resistivity (0.143 ± 0.009 Ω∙m) compared to those printed at 0.20 mm (0.133 ± 0.003 Ω∙m). For the 0.20 mm layer thickness, measurements were conducted on six different track heights: 0.5 mm, 1.5 mm, 3 mm, 5 mm, 7 mm, and 10 mm. For the 0.08 mm layer thickness, three track heights (0.5 mm, 1.5 mm, and 3 mm) were evaluated, producing a set of 18 measurements, as described in Section 2.3. Regarding the resistivity differences between the top and bottom layers, the same pathways, as in the previous tests, with a 0.20 mm building layer thickness, demonstrate a 1% difference with a 1.6% standard deviation, based on 36 measurements taken at different locations. Thus, the maximum 2.6% resistivity variation observed in the conductive specimens with a 0.20 mm building layer thickness (Figure 2a,b) can be considered insignificant, indicating the homogeneity of the electrical characteristics of the fabricated pathways.

Based on the prototype geometry [47], the maximum cross-sectional area of the conductive pathway was chosen to be 2 × 3 mm^2^. The resistivity analysis above determined a layer thickness of 0.20 mm for further calculations and prototyping, balancing conductivity, printing speed, and integration constraints with the drone structure.

Table 4 summarizes the active circuit measurement results for the 25 mm and 77.2 mm conductive pathways with bolted wire connections (Section 2.3 and Figure 6), validating the resistivity findings under functional conditions. The voltage drops across the battery, LED, and conductive tracks were recorded under operating conditions. The measured currents of the 25 mm and 77.2 mm pathways were 1.30 mA and 0.45 mA. Based on these values, the total resistances of the 25 mm and 77.2 mm conductive pathways (including both the printed tracks and the contact resistance at the bolted connections) were found to range from 600 Ω to 615 Ω and from 1756 Ω to 1833 Ω, respectively.

Subsequent four-probe measurements of the disassembled tracks enabled the determination of intrinsic track resistance and resistivity, with the results detailed in Table 4. The resistivity values obtained during the first analysis stage (0.133 ± 0.003 Ω∙m) are consistent with the results in Table 4 (for the 77.2 mm specimen), differing by approximately 1%. The 25 mm specimens exhibit a slightly lower resistivity, which may be attributed to the insufficient measurement length, thereby amplifying the effect of measurement inconsistency. Therefore, further analysis utilizes the results of specimens 77.2 mm long.

The total resistance of the tracks in Table 4 was measured on the activated circuit (e.g., Figure 6b) between the “d” and “e” and “f” and “g” points. The four-probe method measured the track resistance, as shown in Figure 6c. The contact resistance is the calculated difference between the total resistance and the track resistance. Thus, the average contact resistance was estimated to be approximately 71.1 ± 11.0 Ω per track, resulting in 35.6 ± 5.5 Ω per single bolted contact.

### 3.2. Determining the Maximum Length of Conductive Pathway

The calculations are based on a single Li-ion battery type 18650 and four Flywoo RB 11500 KV brushless motors operating at 50% and 100% throttle, to determine the maximum resistance of the 3D-printed conductive pathway. These calculations employ Ohm’s Law for a series circuit, as shown in the schematic diagram in Figure 7a. Since the conductive track and the active component are connected in series with the power source, the total resistance is the sum of the resistance of both connected parts. The available voltage and current requirements of the selected components determine the maximum circuit resistance. The equipment characteristics (Section 2.3) and the measurement results of the conductive tracks (Section 3.1) define the minimum possible conductivity (or the maximum length) of the conductive pathway required to ensure the fabricated prototype is functional. The total resistance (*R*), current (*I*), and voltage (*V*) of the circuit shown in Figure 7a are given as

(3) R=U·I;

(4) $$I=I_{1}=I_{2}=I_{3};$$

(5) $$U_{1}=U_{2}+{\;U}_{3};$$

(6) $$R=R_{2}+R_{3};$$

(7) $$R_{2}=U_{2}/I_{2}=\left(U_{1}-U_{3}\right)/I_{3},$$

where the subscripts “1”, “2”, and “3” correspond to the notations in Figure 7.

The maximum achievable length of the 3D-printed conductive track is calculated based on the resistance value determined in Section 3.1. The calculations pertain to the LED active component, excluding the motor, and utilize the measurement results of the conductive pathways with a 0.20 mm layer thickness (Figure 2a,b). The conductive pathways (Figure 5) exhibit an average resistivity of 0.133 ± 0.003 Ω∙m. The length is calculated using Ohm’s Law based on the nominal LED values (a forward voltage between 2.2 V and 2.5 V, a nominal current of 2 mA) [48] and the measured resistivity of the pathway (Figure 5). Thus, the calculations assume an average voltage of 2.35 V and a resistivity of 0.133 Ω∙m. The following formula determines the maximal length of the conductive pathway

(8) $$l_{max}=\frac{R\cdot A}{\rho }=\frac{\left(U_{1}-U_{3}\right)\;\cdot \;A}{I_{3}\;\cdot \;\rho }=\frac{\left(3.6-2.35\right)\;\cdot \;2\;\cdot \;3}{2\;\cdot \;10^{-3}\;\cdot \;133}=28.2\;mm,$$

where *R* is the conductive track resistance calculated using Equation (7), simple calculations determined that the maximum possible conductive length varied from 4.4 mm for a 2 × 0.5 mm^2^ cross-section area to 88.1 mm for the maximum 2 × 10 mm^2^ cross-section area of the conductive pathway considered in this study. However, based on the geometry of the Nano Long Range drone, the minimum track length required per arm is 206.4 mm.

The 2 × 3 mm^2^ cross-section of the conductive pathway is the maximum allowable by the drone geometry (Section 2.5). Therefore, this analysis also uses this cross-section size to determine the maximum pathway’s length. Based on the estimated conductivity, this cross-section geometry allows a maximum track length of 28.2 mm. However, for safety reasons (accounting for potential printing imperfections), this value is reduced by 12%, resulting in a final design length of 25 mm, which ensures consistent performance in practical applications. This approach provides a realistic and reliable conductive track design, optimizing both electrical and structural considerations for integration into nano drone circuitry.

### 3.3. The Working Circuit Example

**Figure 11:**
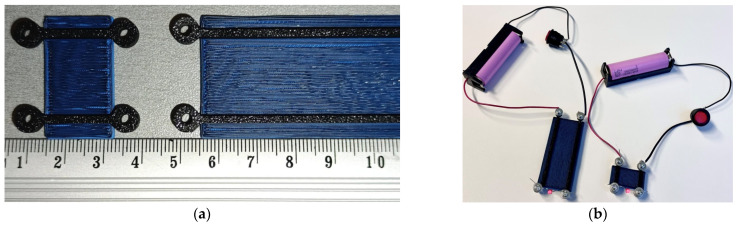
Electrical circuits with conductive tracks with bolted wire connections: (**a**) 3D-printed specimens; (**b**) activated 25 mm and 77 mm circuits with lighting LEDs.

As detailed in Table 5, the experimental setup consisted of a single Li-ion battery type 18650 (3.6 V, 3450 mAh) housed in a holder, a momentary push-button switch, an LED with an operating current of 2 mA, and conductive wires. The 3D-printed conductive tracks (Figure 11a), fabricated utilizing the printing parameters specified in Section 2.2, were integrated into the circuit, replacing a segment of the conventional conductive wiring, as shown in Figure 11b. The bolted connection method ensured a stable interface between the printed tracks and metal wiring, resulting in minimal contact resistance.

Initially, a 25 mm conductive track was fabricated, following the calculations outlined in Section 3.2 and employing an arbitrary safety factor of 1.136. After the active component was activated (illuminating the LED, Table 5), a second track of 38.6 mm—the maximum length calculated for the nominal LED current—was printed and tested. Since this configuration also functioned, a third track of 77.2 mm (twice the maximum calculated length) was fabricated to assess whether the LED could operate at a reduced current. The trial (Figure 11b) confirmed that the LED remained functional at currents below its nominal value. Thus, the bolted connections have proved to be reliable.

**Figure 12:**
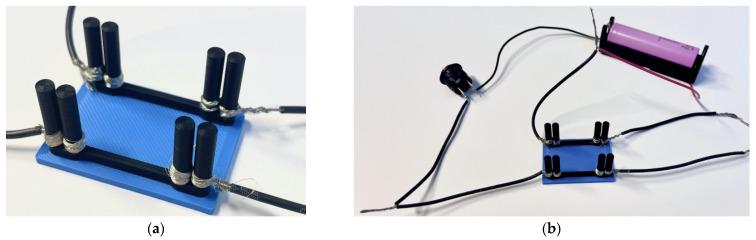
Electrical circuits with conductive tracks with wire-wrapping connections: (**a**) wire-wrapping connection; (**b**) activated circuit.

An alternative connection method was tested to evaluate the feasibility of 3D-printed conductive tracks, utilizing wire-wrapping around 3D-printed posts (Figure 3b). The experimental setup remained consistent with the first bolted connection type, with identical printing parameters and the same circuit components listed in Table 5. However, instead of securing the conductive wires with bolts, they were wrapped in a figure-eight pattern around circular 3D-printed posts, as shown in Figure 12a. Like the bolted samples, the conductive (black) pathways were formed on the ordinary PLA (blue) plate. This design aimed to eliminate the need for additional hardware, such as bolts, nuts, and washers, thereby reducing the overall weight of the circuit, a crucial factor for drone applications.

Tightly wrapping the wire around the posts without the use of solder or external fasteners established a mechanically and electrically stable connection. When the circuit was assembled and powered, the LED illumination (Figure 12b) demonstrated that the electrical performance of the wire-wrapping was comparable to that of the bolted connection. These results indicate that wire-wrapping is a viable, lightweight alternative for integrating 3D-printed conductive tracks into electronic circuits while maintaining reliable conductivity.

### 3.4. The Heating Effect on the Conductivity of the Printed Circuit

The 25 mm long specimens were exposed to 60 °C for an hour in a heating chamber to assess the thermal stability and residual conductivity of 3D-printed conductive tracks. Three specimens of both connection types (i.e., Figure 11a and Figure 12a) were tested. Before heating, the initial resistance of each specimen was determined. The measurements were repeated twice: (1) immediately after the heating and (2) after cooling to room temperature for an hour. Table 6 summarizes the measurement results. In this table, the letter “B” designates the bolted connection and “W” corresponds to the wrapped wire connection; the numbers differentiate the identical specimens in the test series.

All the tested specimens exhibited a notable increase in resistance immediately after being subjected to 60 °C. This increase may result from the thermal expansion of the polymer matrix [20]. As the polymer expands, conductive pathways become more dispersed, increasing electrical resistance. Although the resistance decreased somewhat upon returning to room temperature, it remained consistently higher than the initial measurements in both connection types. This permanent increase in resistance may be attributed to microstructural changes—minor microcracks or voids could form within the conductive polymer matrix during thermal expansion and contraction, impairing the continuity of conductive pathways, as well as interfacial or contact degradation—even with bolts or wire-wrapping, surface oxidation, relaxation of contact pressure, or partial delamination might occur at the interface, further increasing contact resistance. Further analysis employs the bolted connection due to its stability and the less scattered results of the reference specimens (compared to their wrapped counterparts) presented in Table 6.

### 3.5. The Current-Induced Heating of the Printed Specimens

**Figure 13:**
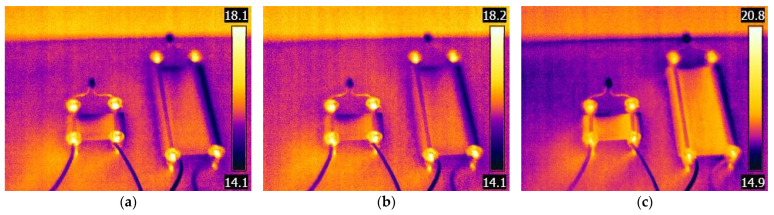
Thermal distribution images of the circuits operating at 15.0 °C captured after (**a**) 10 min.; (**b**) 30 min.; (**c**) 70 min. of activation. Note: The color bars indicate the temperature in °C.

This test assesses whether a 3D-printed conductive track generates heat during a regular circuit operation and whether such heating could impact circuit performance or compromise the mechanical integrity of the printed structures. Section 2.4 describes that two 25 mm and 77.2 mm long conductive tracks with bolted wire connections were evaluated under controlled thermal conditions. Figure 13 shows the selected temperature distributions captured using the FLIR E60BX camera (Teledyne FLIR, Wilsonville, OR, USA).

Figure 13 illustrates the approximately 3 °C to 6 °C increase in temperature of the conductive specimens resulting from electrical conduction, given an environmental temperature of 15.0 °C. The point temperature measurement results ensured by FLIR BuildIR software (2.11.0315, Teledyne FLIR, Wilsonville, OR, USA) demonstrate a near 10 °C temperature increase in the 25 mm long specimen; in the 77 mm specimen, this alteration slightly exceeds 7 °C. However, the temperature increase is localized at the bolted contacts, and the conductive pathways do not exhibit substantial temperature changes. The highest temperature recorded on the conductive pathways was 18.3 °C, i.e., 3 °C higher than the environmental temperature. Therefore, the electric current barely induces the heat required to reach the glass transition temperature of ProtoPasta [31], which varied from 58.9 °C to 63.3 °C. This result indicates the neglect of electricity-induced temperature alterations for the selected battery type. Additionally, the prototyping should avoid wires and the corresponding contact problems associated with sudden conductivity changes [49,50].

### 3.6. Estimating the Minimal Polymer Conductivity

Table 4 presents all the measured and calculated electrical characteristics necessary for estimating the average contact resistance at the bolt and track interface. The average contact resistance (per four bolted contacts) is estimated to be 142.4 ± 22 Ω per circuit, based on the measurement data for both conductive lengths. The following expression determines the maximum resistance of the conductive track:

(9) $$R_{2,max}=R-R_{3}-\sum R_{4}\;=\frac{U_{1}-U_{3}}{I}-\sum R_{4},$$

where *R*, *U*, and *I* represent the circuit’s total resistance, voltage, and current. The subscripts “1”, “2”, “3”, and “4” correspond to the battery, 3D-printed track, active circuit (LED), and the bolted contact. The current and voltage values were obtained from the configuration with the 77.2 mm track presented in Table 4. This setup provided a more representative basis for determining the upper resistance limit of the conductive track and, by extension, its maximum practical length. Thus, the resultant track resistance is 4.08 kΩ.

Rearranging Equation (8), the maximum length of the printed conductive pathway can be expressed as

(10) $$l_{max}=\frac{R_{2,max}A_{2}}{\rho_{2}}=\frac{4080\;\cdot \;2\;\cdot \;3}{131.2}=186.6\;mm,$$

where *ρ*_2_ (=0.1312 Ω∙m) is the average resistivity in Table 4.

Assuming the 2 × 3 mm^2^ cross-section, 4.08 kΩ resistance, and 0.1312 Ω∙m resistivity of the conductive track, Equation (10) results in the 186.6 mm maximum length of the conductive pathway, which theoretically ensures the LED operation in the conditions considered. This result represents the sum of all the printed fragments in the circuit. For example, the samples, shown in Figure 11 and Figure 12, consist of two 3D-printed tracks. This estimated length is almost seven times greater than the results of Section 3.2, due to less demanding LED operation conditions than those specified by the supplier [48]. In particular, the voltage decreased 1.4 times, from 2.35 V to 1.66 V, and the current decreased 4.4 times, from 2 mA to 0.45 mA.

### 3.7. Analyzing Functionality of the Nano-Drone Prototype

**Figure 14:**
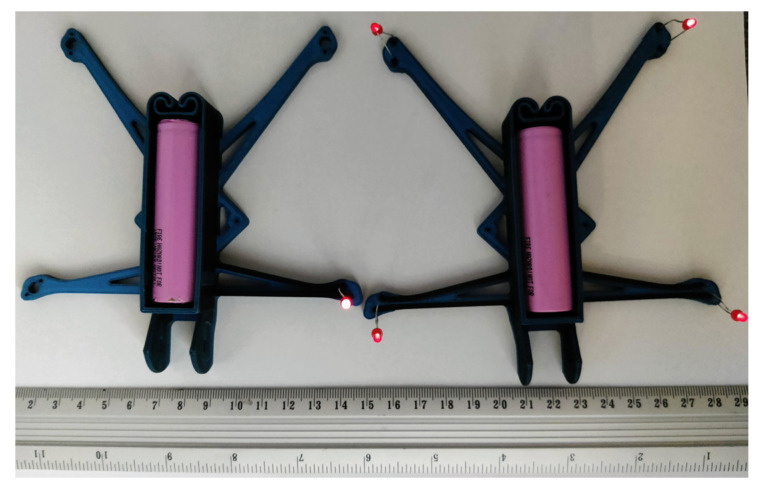
The working examples of wireless drone prototypes, incorporating lit LEDs, conductive polymer pathways, and a battery holder, are shown in Prototype 1 (**left**) and Prototype 2 (**right**).

As determined in Section 3.2, the inherent resistance of the available conductive polymer (ProtoPasta [31]) is too high to replace conventional wiring in high-current applications. Consequently, this study replaced the drone’s engines with LEDs to serve as a low-power load, thereby mitigating the impact of the polymer’s limited conductivity (Section 2.5). Furthermore, a battery holder was custom-designed and fabricated using the conductive polymer; LEDs were tightly installed into the pre-fabricated holes. This means eliminating metal elements from the drone’s electrical system. Figure 14 shows the working examples of Prototypes 1 and 2, featuring lit LEDs and metric (mm) and imperial (inch) scales to facilitate estimation of the model’s geometry dimensions. The brightness of the single LED (Prototype 1) outperforms the light intensity of four LEDs in Prototype 2 due to current limitations. Still, even though the LEDs are not as bright, the fully printed Prototype 2 remains functional. This approach highlights the potential of additive manufacturing in reducing weight and complexity, while demonstrating a novel methodology for producing integrated, fully 3D-printed circuits.

Further analysis determines the conductivity characteristics, which ensure the full functionality of the 3D-printed prototype of the Nano Long Range drone [47]. This condition encompasses the fully printed structure of the drone, including its conductive network, as well as its flight capabilities. The drone geometry and existing equipment (Section 2.5) determine the length and cross-sectional area of the embedded conductive tracks. The calculations focus on the conductive pathways from the battery to the four Flywoo RB 11,500 KV brushless motors, operating at 50% and 100% throttle. A nominal 5 V power source is assumed to simplify analyzing voltage drop and current flow within the drone’s geometry. Table 7 summarizes the current and voltage values. Theoretical calculations determine the polymeric material’s minimum conductivity (or the maximum permissible resistivity) to ensure a sufficient current supply to each motor without incurring significant power losses or performance degradation.

The ratio of the track voltage (*U*_2_) and current (*I*_2_) determines the maximum track resistance (*R*_2,*max*_). At 50% and 100% throttle, the maximum resistance equals 1.130 Ω and 0.265 Ω, respectively. The SolidWorks model (Figure 8b,c) determines the geometry characteristics of the conductive pathways listed in Table 8.

The following expression determines the maximum resistivity of the tracks

(11) $$\rho_{2,max}=\frac{R_{2,max}\cdot A_{2}}{l_{2}}=\frac{R_{2,max}}{\sum l/A},$$

where the denominator represents the summation of all geometry fragments belonging to the arm in Table 8. The parallel circuit scheme of the drone prototype (Figure 7b) ensures that only the back arm is considered for the maximum resistivity calculations due to its longer geometry. This table also includes the maximum resistivity calculation results.

Since conductivity (*σ*) is the inverse value of resistivity (Equation (2)), the results of Table 8 determine the minimum 27.24 × 10^3^ S/m conductivity of the polymeric pathway at 50% throttle. At 100% throttle, the minimum conductivity demands reach 116.1 × 10^3^ S/m, substantially exceeding the available characteristics (16.7 × 10^3^ S/m) in Table 2. Considering the literature results (Table 1 and Table 3), only high-volume content of the silver particles (55 wt%) utilized by Lei et al. [30] ensures the acceptable 142 × 10^3^ S/m conductivity of the polymeric pathway.

However, high metal filler content may also compromise the mechanical and thermal properties of the printed polymeric components, reducing the material’s strength and ductility and harming the drone’s structural integrity [51,52,53]. Furthermore, thermal management remains vital, as localized heating from higher current densities may degrade electrical performance and mechanical stability [49].

### 3.8. Future Research

To quantify the performance of the 3D-printed conductive paths, the study employed a multi-stage evaluation focusing on electrical resistivity, thermal stability, and current-induced heating, with particular attention to the constraints imposed by the application in drone systems. The previous tests [3] revealed that the strength of PLA is sufficient for drone applications, significantly exceeding the design stress values by nearly 30 times. However, long-term resistance of polymeric materials raises substantial concerns [54]. The long-term conductivity stability of CP is also low [6,55].

When operating drones, external and operational temperature changes, vibrations from high-speed rotating propellers, air turbulence, and the contact force with the landing surface influence the mechanical resistance of drone materials. The engine heats significantly, and the conductor through which strong currents flow also heats up, resulting in electromagnetic interference (EMI). Prolonged exposure to sunlight over several months causes the polylactic acid to degrade. As a result, the surface quality deteriorates, impacting the cross-sectional area of the printed conducting pathways and channels. Therefore, future designs should incorporate a sunlight-reflective surface to reduce the absorption coefficient in the UV and visible spectra and account for the long-term deterioration of the physical performance of polymeric materials.

The analysis of the results obtained in this study shows that replacing metal wiring in a drone’s primary power system with 3D-printed conductive polymers is technically feasible, provided the material’s conductivity meets the calculated minimum threshold of 116.1 × 10^3^ S/m. Regarding wire-based electricity supply systems, CP materials are unlikely to achieve the conductivity characteristics of pure metals. For instance, copper and aluminum, typically used for wires, possess conductivities of 59.6 × 10^6^ S/m and 35.0 × 10^6^ S/m, which are nearly 250 to 420 times higher than the target value in this study. Copper and aluminum have densities of 8940 kg/m^3^ and 2700 kg/m^3^, which are 2 to 7.2 times higher than the density of PLA (1240 kg/m^3^). However, in the case of an electromagnetic pulse (EMP) generated by an electromagnetic gun, a wide cross-sectional conducting path integrated into the drone body is more reliable for handling high-power electromotive forces [56,57]. This effect results from the relatively small diameter of a metal wire, which experiences significantly higher current densities and therefore cannot act as a current-limiting component as effectively as a PLA pathway embedded with conductive nanoparticles.

Furthermore, the CP for FFF reduces the need for manual wire installation for electrical equipment. This fabrication enhancement reduces the need for manual assembly, minimizes errors associated with wiring electronic components, and minimizes manual interruptions to the manufacturing process, making it autonomous and adaptable for various industrial needs. The manufacturing of raw polymeric materials is also less equipment-demanding and much more flexible than metal wiring fabrication. Thus, materials science and AM advancements could enable fully integrated, lightweight, and functionally optimized electrical systems directly embedded into the drone’s structure. However, manufacturing the pathways (Figure 10b) requires nearly 10 g of conductive polymeric material. The CP may cost 20 EUR per drone, accounting for market prices (Table 2). Optimizing the printed circuit structure in Figure 10 and increasing the material’s conductivity may reduce financial expenses. Therefore, the 2000 EUR/kg price sets the target for further development of the CP material.

## 4. Conclusions

Evaluating conductive polymers as a viable substitute for metal wiring in drones necessitates identifying the minimum electrical conductivity for successful integration. The characterization of conductive polymeric materials available on the market, along with a simplified redesign of the Nano Long Range drone’s frame to incorporate conductive circuits, replace all conventional wiring, and ensure it is fully 3D-printable, exemplifies the design concept considered. This analysis identifies the performance thresholds a conductive polymer must meet to ensure reliable operation, focusing on the primary power delivery path (from the battery to the motors). The following essential conclusions have emerged from this study:

- The four-point probe method is confirmed to be a reliable technique for evaluating the electrical resistivity of 3D-printed conductive pathways, minimizing the influence of contact resistance.
- The analysis of the conductive polymeric filaments for 3D printing determines the cost efficiency of the carbon black (CB) additives for prototyping purposes. On the contrary, the graphene additives substantially increase the material costs, making them comparable to metal particle-modified alternatives, but do not significantly enhance the conductivity of polymeric materials.
- Both bolted and wrapped wire contacts, considered in this study, ensure comparable electrical efficiency suitable for prototyping. However, these joints introduce uncertainty into the circuit due to the increased scatter of contact properties (resistance) and cause heating within the electrical circuit. Further prototypes should avoid the wire contacts to ensure the circuit’s reliability.
- The increased temperatures (around 60 °C) reduce the conductivity of the 3D-printed circuits, resulting in a residual decrease in electrical performance. Depending on the contact type (i.e., bolted or wrapped), the residual resistance increases from 36% to 66% after heating the specimens at 60 °C and cooling them under laboratory conditions. These temperature conditions are common and should be considered when designing polymeric circuits.
- The operation conditions considered in this study, i.e., replacing motors with LEDs, do not induce dangerous heating of the conductive polymeric pathway. The maximum temperature increase of 10 °C was observed in the relatively short specimens, which were 25 mm long. This temperature increase is localized at the bolted contacts, and the conductive pathways do not exhibit substantial temperature changes. Still, increased voltage will increase the heating effect in real-world applications. Therefore, the polymeric circuit design should avoid the wires and corresponding contact problems associated with the sharp changes in conductivity within the circuit.
- The conductivity characteristics analysis, ensuring the full functionality of the 3D-printed Nano Long Range drone prototype, determines the minimum 116.1 × 10^3^ S/m conductivity of the polymeric pathway, which substantially exceeds the market’s available characteristics of the conductive polymeric materials, with the maximum value of 16.7 × 10^3^ S/m. Considering the literature results collected in this study, only a high volume content of silver particles (55 wt%) ensures the acceptable 142 × 10^3^ S/m conductivity of the polymeric pathway. Still, the cost of the conductive polymer should not exceed 2000 EUR/kg to remain competitive on the market. These findings inform material criteria and guide future development of functional, fully integrated 3D-printed electronic systems in drone applications.

## Figures and Tables

**Table 1:** * Conductivity of FFF polymers.

Ref.	Matrix	Conductive Filler	Conductivity [S/m]	Application Potential
[20]	PP	CB (32.3 wit%)	0.10…0.50	Conductive circuits and sensors
[26]	TPU	MWCNT (5 wt%)	1	Strain sensors
[27]	PLA	MWCNT (10 wt%)	100	Structural and conductive components
[28]	PMMA + PPy (10 wt%)	Graphene (10 wt%)	1.42 × 10^3^	Wiring and printable conductive inks
[29]	PA6	Nickel particles (dispersed with Sn_95_Ag_4_Cu_1_) (30 vol%)	31.0 × 10^3^	Conductive tracks
[30]	PVB	Silver particles (55 wt%)	142 × 10^3^	PCB, RFId, and electronic paper

* PP = polypropylene; TPU = thermoplastic polyurethane; PLA = polylactic acid; PMMA = polymethyl methacrylate; PPy = polypyrrole; PA6 = polyamid 6; PVB = polyvinyl butyral; MWCNT = multi-walled carbon nanotubes; PCB = printed circuit board; RFId = radio frequency identifier.

**Table 2:** * Commercially available conductive polymer 1.75 mm filaments.

Ref.	Matrix	Filler	Conductivity [S/m]	Cost * [€/kg]	Application Potential
[31]	PLA	CB	3…7	85.22	Low-current applications, wearable electronics, capacitive touch sensors
[32]	PLA	CB	4	112.49	Low-current applications, anti-static parts, and EMI and RFI shielding
[33]	PLA	CB	70	99.75	Low current applications and capacitive touch sensors
[34]	TPU	G	80	759.50	Low-current applications, capacitive touch sensors, wearable electronics, medical devices, neural surface electrodes, and EMI and RFI shielding
[35]	PLA	G	167	1990.00	Capacitive touch sensors and conductive circuitry for electronics
[36]	PL	Copper particles	16.7∙10^3^	2035.94	Electrically conductive circuitry for electronics and EMI and RFI shielding

* PLA = polylactic acid; TPU = thermoplastic polyurethane; PL = polyester; CB = carbon black; G = graphene; EMI = electromagnetic interference; RFI = radio frequency interference. ^⁑^ Costs collected in February 2025 for the delivery to Vilnius, Lithuania.

**Table 3:** Test results of the conductive FFF sample prototypes.

Ref.	Printer	Cross-Section [mm × mm]	Printing Base	Matrix *	Filler	Conductivity [S/m]
[20]	Makerbot Dual and FlashForge Creator	0.4 × 0.2	ABS	PP	CB	200
[38]	Makerbot Replicator 2	0.8 × 0.1	PI film	PLA	Graphene	476
[39]	Customized D-Bot	0.5 × 0.4…1.0	Painter’s tape	PLA	CB	8
					Graphene	128
				PL	Copper particles	7.1 × 10^3^

* ABS = acrylonitrile butadiene styrene; PI = polyimide; PP = polypropylene; PLA = polylactic acid; PL = polyester.

**Table 4:** Electrical characteristics of the conductive tracks with the bolted wire connections.

Length [mm]	Current [mA]	Voltage [V]			Resistance [Ω]			Resistivity [Ω∙m]
		Battery	LED	Track	Total	Track	Contact	
25 *	1.30	3.58	1.74	0.78	600 ± 2	540 ± 20	60 ± 18	0.1296 ± 0.0048
				0.80	615 ± 1	530 ± 30	85 ± 29	0.1272 ± 0.0063
77.2 *	0.45	3.56	1.66	0.79	1756 ± 3	1690 ± 40	66 ± 37	0.1313 ± 0.0087
				0.83	1833 ± 3	1760 ± 50	73 ± 43	0.1368 ± 0.0110

* Two rows corresponding to the same specimen length represent different conductive tracks of the sample (e.g., Figure 4).

**Table 5:** The circuit components.

Type	Specification	Quantity
Battery	Li-ion type 18650, 3.6 V, 3450 mAh, 8 A	1
Battery holder	Holder with wires	1
Active component	LED Ø 3 mm type L-934LID, 2 mA (red)	1
Switch	Latching switch M/R13-523FR	1
Wire	Silicone tinned copper 28-core wire (26AWG)	As required
Bolt ^1^	M2 × 12 mm galvanized steel	4
Nut ^1^	M2 galvanized steel	4
Washer ^1^	M2 galvanized steel	8

^1^ Used for bolted connections only.

**Table 6:** Resistance of the heated specimens at 60 °C [Ω].

Specimen	Reference	Heated	Cooled
B1	708	1240	1026
B2	731	1625	1311
B3	647	1227	1112
Mean ± Std. Dev.	695 ± 43	1364 ± 226	1150 ± 146
Difference	–	96.3%	65.5%
W1	998	1710	1240
W2	795	1142	993
W3	896	1556	1430
Mean ± Std. Dev.	896 ± 102	1469 ± 294	1221 ± 219
Difference	–	64.0%	36.3%

**Table 7:** Electrical characteristics of the drone prototype with four motors.

Item	Voltage, *U* [V]	Current, *I* [A]	
		At 50% Throttle	At 100% Throttle
Motor Flywoo RB 11500KV	3.7	1.15	4.9
Printed track (per arm)	1.3	1.15	4.9
Drone (total)	5.0	4.6	19.6

**Table 8:** Geometry parameters of the conductive tracks.

Arm	Area, *A* [mm^2^]	Length, *l* [mm]	Resistivity, *ρ* [μΩ∙m]	
			At 50% Throttle	At 100% Throttle
Front	6	149.4	41.25	9.681
	20	41.0		
	30	6.0		
	40	10.0		
Back	6	171.1	36.71	8.617
	20	35.2		
	30	8.0		
	40	10.0		

## Data Availability

Data are contained within the article.
